# Angle-of-Arrival Assisted GNSS Collaborative Positioning

**DOI:** 10.3390/s16060918

**Published:** 2016-06-20

**Authors:** Bin Huang, Zheng Yao, Xiaowei Cui, Mingquan Lu

**Affiliations:** Department of Electronic Engineering, Tsinghua University, Beijing 100084, China; b-huang09@mails.tsinghua.edu.cn (B.H.); cxw2005@tsinghua.edu.cn (X.C.); lumq@tsinghua.edu.cn (M.L.)

**Keywords:** GNSS, angle-of-arrival, Cramer-Rao lower bound, collaborative positioning

## Abstract

For outdoor and global navigation satellite system (GNSS) challenged scenarios, collaborative positioning algorithms are proposed to fuse information from GNSS satellites and terrestrial wireless systems. This paper derives the Cramer-Rao lower bound (CRLB) and algorithms for the angle-of-arrival (AOA)-assisted GNSS collaborative positioning. Based on the CRLB model and collaborative positioning algorithms, theoretical analysis are performed to specify the effects of various factors on the accuracy of collaborative positioning, including the number of users, their distribution and AOA measurements accuracy. Besides, the influences of the relative location of the collaborative users are also discussed in order to choose appropriate neighboring users, which is in favor of reducing computational complexity. Simulations and actual experiment are carried out with several GNSS receivers in different scenarios, and the results are consistent with theoretical analysis.

## 1. Introduction

With the development of global navigation satellite systems (GNSS) and wireless communication technologies in recent years, GNSS collaborative positioning is becoming attractive [1]. Nowadays, there are many scenarios in which multiple GNSS receivers work simultaneously in a certain region [2]. For instance, a platoon of military men carries out a task together, people locate their positions in a shopping center, *etc*. The collaboration between GNSS receivers refers to a group of users conducting joint data processing by sharing the measurements from GNSS signals and certain relative measurements (distance, azimuth or elevation) with their neighboring users. Collaborative localization has been an active research topic, because it provides several potential advantages, including better positioning availability, stability and accuracy. In challenging environments, such as urban canyons and forests, GNSS standalone receivers usually fail to provide reliable positioning results. However, GNSS collaborative positioning can be a promising method.

In the previous studies on GNSS collaboration, only the relative distance measurements between neighboring users were involved. The collaboration between GNSS users was first proposed in [3] by using the weighted least squares (WLS) algorithm, and then, the estimation algorithms based on the Kalman filter (KF) have been used to solve the collaborative localization problem [4,5,6]. In [7], the Cramer-Rao lower bound (CRLB) for collaborative positioning with relative distance measurements is derived, and the effects of various factors are also analyzed and verified based on the collaborative dilution of precision (CDOP) model in [8].

Compared to localization with only distance measurements, the localization can also be performed using angle-of-arrival (AOA) measurements [9]. In general, location estimates of mobile users are derived from two types of measurements: AOA and range. However, specific algorithms and analysis on GNSS collaborative positioning with AOA measurements between neighboring users are still lacked. The AOA measurements are usually achieved by applying antenna arrays. There are either iterative or non-iterative AOA positioning methods proposed in [10,11,12]. However, all of them only employ the AOA measurements from the anchor users and have ignored the interconnectivity among the neighboring blind users. For the collaboration with relative AOA measurements, the researchers of [13] investigate the nonlinear observability of collaborative localization for two robots, and the analysis shows that relative bearing is the best observation between the robots. In [14], the cooperative positioning methods with the AOA measurements in wireless sensor networks are proposed, and the cooperative localization simulation and experimental results are shown in [15].

In this paper, a new technique is proposed that makes use of AOA measurements in conjunction with GNSS collaborative positioning. It assumes that the AOA measurements with neighboring users are available for the collaborative user group. Then, the CRLB of different collaborative positioning and statistical analysis of positioning errors are deduced. In GNSS standalone positioning, each user only obtains the pseudo-range measurements from its visible GNSS satellites; however, in GNSS collaborative positioning, the available measurements include extra measurements (distances, azimuths or elevations) between the neighboring users. As a result, there are more impact factors on the collaborative positioning accuracy, which brings more problems to the process of performance analysis. Based on the derivation of CRLB and positioning algorithms, the comparison with GNSS positioning without AOA measurements is performed by theoretical analysis. The effects of various factors are also analyzed and verified, such as the impacts of the number of collaborative users, their distribution and AOA measurement accuracy. In addition, considering that there are some limitations on increasing the number of users due to power and complexity constraints, the influence of the relative location of the collaborative users is also discussed in order to choose appropriate neighboring users.

Finally, in outdoor and partially-blocked simulation scenarios, respectively, simulation tests are carried out with several GNSS users. The simulation results fall in line with the results of theoretical analysis. Besides, the collaborative positioning experiment is carried out with several GNSS receivers in an actual scenario. The conclusions provide useful information for the design and test of actual collaborative applications.

The remainder of this paper is organized as follows. Section 2 gives a brief overview of the collaborative positioning signal and system model. Then, in Section 3, expressions for the Fisher information matrix (FIM) and CRLB are derived for different collaborative cases. The collaborative WLS positioning algorithm is proposed in Section 4. Based on this CRLB model, Section 5 presents the theoretical analysis. The impacts of various factors are included, and the comparison with GNSS standalone positioning is done. In Section 6, the simulation tests are carried out to verify the analysis results in different scenarios. The results of the experiment in an actual scenario and discussions are presented in Section 7. Finally, the conclusions are given in Section 8.

## 2. Signal Model

**Figure 1:**
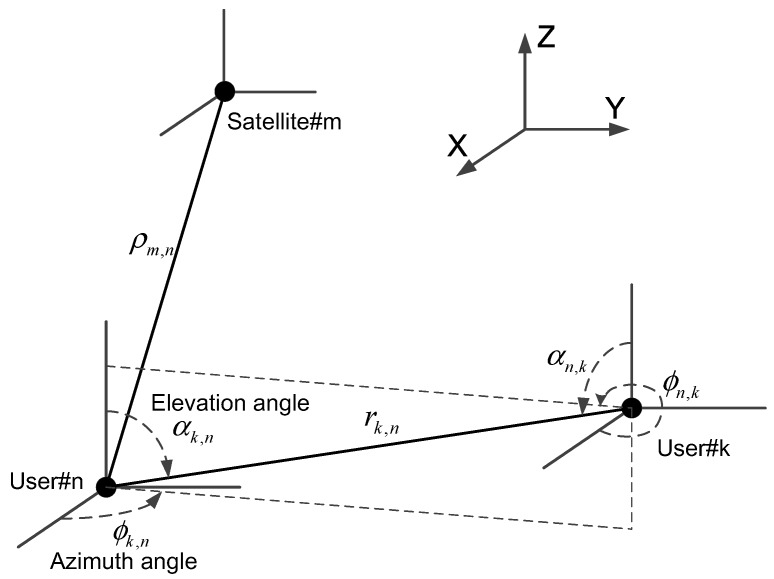
Three-dimensional satellites and user distribution.

Consider a collaborative network in which there are *N* users, each of which is equipped with a GNSS receiver and is able to obtain relative measurement estimates from its neighboring users. The relative measurements usually contain distance and AOA (both azimuth and elevation angles) estimates. Figure 1 is a simple diagram constructed to provide a mental image and understanding of GNSS collaboration. As shown in Figure 1, user #*n* and user #*k* are within the radio range of each other.

In this paper, the collaborative positioning algorithm is a centralized algorithm, and all of the measurements are processed in a collaborative process center. After the execution of the positioning algorithm, the process center sends the positioning results to all of the users. In most cases, the collaborative process center is one of the users. For example, the user #1 can be chosen as the process center in Figure 1. Of course, a dedicated process center can also be used.

For any user #*n* in the collaborative network, the three-dimensional position and clock error expressed in the distance unit are indicated respectively by:

(1) $$\begin{array}{c} p_{n}^{u}={\left[x_{n}^{u},y_{n}^{u},z_{n}^{u}\right]}^{T},\;b_{n}^{u}=c\times \delta t_{n}^{u},\;n=1,2,...,N \end{array}$$

where c is the speed of light. Denote by $M_{n}$ the set of the visible GNSS satellites and by $K_{n}$ the set of collaborative users for user #*n*. The three-dimensional positions of visible satellites and collaborative users are indicated respectively by:

(2) $$\begin{array}{c} p_{m}^{s}={\left[x_{m}^{s},y_{m}^{s},z_{m}^{s}\right]}^{T},\;m\in M_{n} \end{array}$$

and:

(3) $$\begin{array}{c} p_{k}^{u}={\left[x_{k}^{u},y_{k}^{u},z_{k}^{u}\right]}^{T},\;k\in K_{n} \end{array}$$

For GNSS collaboration in this paper, there are three kinds of measurements that can be used.

### 2.1. Pseudo-Range Measurements

In general, for GNSS positioning, the users cannot directly measure the real ranges to the satellites, but determines the estimate of pseudo-ranges from the GNSS tracking process. The pseudo-range measurement is defined as the sum of the propagation delay from the receiver to the satellite plus the unknown clock bias, which is given by:

(4) $$\begin{array}{c} \rho_{m,n}=d_{m,n}^{s}+b_{n}^{u}+n_{m,n}^{\rho },\;m\in M_{n} \end{array}$$

where $d_{m,n}^{s}=∥p_{m}^{s}-p_{n}^{u}∥$ is the real range from satellite #*m* to user #*n* and $n_{m,n}^{\rho }$ is the measurement noise of the tracking process. $n_{m,n}^{\rho }$ is typically made up of satellite ephemeris and clock error, atmospheric (ionosphere and troposphere) transmission error, receiver thermal noise, multipath error, *etc*. [16].

### 2.2. Distance Measurements

In addition to using pseudo-range measurements, GNSS collaborative positioning algorithms may use the relative distance measurements between the neighboring users. There are many kinds of measuring methods for the distance measurements, for example by measuring the round-trip time.

For user #*n*, the distance measurement from its neighboring user #*k* is given by:

(5) $$\begin{array}{c} r_{k,n}=d_{k,n}^{u}+n_{k,n}^{r},\;k\in K_{n} \end{array}$$

where $d_{k,n}^{u}=∥p_{k}^{u}-p_{n}^{u}∥$ is the real range of two users and $n_{k,n}^{r}$ is the distance measurement noise. In general, $n_{k,n}^{r}$ is relative to the ranging technology and the external environment. At present, the most common ranging technologies are based on the measurements of received signal strength (RSS) or time-of-arrival (TOA). Typically, meter-level accuracy of relative distance measurements can be achieved, and for some high accuracy ranging technology, like ultra-wideband (UWB) for TOA, centimeter level accuracy can be obtained [17].

### 2.3. AOA Measurements

Most of the existing research on GNSS collaborative positioning are based on the pseudo-range and distance measurements. Specific algorithms and performance analysis with AOA measurements are lacked. In this paper, we add the AOA measurements to GNSS collaborative positioning and analyse the positioning performance with different types of measurements. The AOA collaborative technique is based on the measurement of angles between the collaborative users. Generally, the AOA measurements can be achieved by applying an antenna array.

The azimuth and elevation measurements by user #*n* from user #*k* are given respectively by:

(6) $$\begin{array}{c} \phi_{k,n}={\tilde{\phi }}_{k,n}+n_{k,n}^{\phi },\;\alpha_{k,n}={\tilde{\alpha }}_{k,n}+n_{k,n}^{\alpha },\;k\in K_{n} \end{array}$$

where ${\tilde{\phi }}_{k,n}$ and ${\tilde{\alpha }}_{k,n}$ are respectively the real azimuth and elevation between user #*n* and user #*k*. In the physical sense, ${\tilde{\phi }}_{k,n}$ is the line user #*n* point to user #*k* of the angle with the *x* axis and ${\tilde{\alpha }}_{k,n}$ the line of the angle with the *z* axis. The value of ${\tilde{\phi }}_{k,n}$ ranges from 0° to 360°, and the value of ${\tilde{\alpha }}_{k,n}$ ranges from 0° to 180°. They are defined as:

(7) $$\begin{array}{c} {\tilde{\phi }}_{k,n}=\mathrm{atan}2\left(y_{k}^{u}-y_{n}^{u},x_{k}^{u}-x_{n}^{u}\right),\;{\tilde{\alpha }}_{k,n}=\mathrm{acos}\left(\frac{z_{k}^{u}-z_{n}^{u}}{d_{k,n}^{u}}\right) \end{array}$$

$n_{k,n}^{\phi }$ and $n_{k,n}^{\alpha }$ are respectively the measurement noises of the relative azimuth and elevation. Their noise variance highly depends on the communication environment, the AOA detection device (method) and line-of-sight (LOS) connections. Therefore, the multipath effects, such as reflections, scattering and fading, have a negative influence on positioning performance, and it is very difficult to find a single model that can be applied to all situations [18]. For analytical convenience, a simple model is used to characterize the AOA measurements combining the errors caused by multipath, the channel and the device/method. With the development of the AOA measuring technologies, it can already guarantee that the standard deviations of AOA measurements are always smaller than 10° [10].

## 3. Cramer-Rao Lower Bound

For the user network, define the unknown vector as:

(8) $$\begin{array}{c} \theta ={\left[{\left(p_{1}^{u}\right)}^{T},b_{1}^{u},{\left(p_{2}^{u}\right)}^{T},b_{2}^{u},...,{\left(p_{N}^{u}\right)}^{T},b_{N}^{u}\right]}^{T}\in R^{4N\times 1} \end{array}$$

which contains the three-dimensional position and clock bias of each user. The CRLB sets a lower bound on the variance of any unbiased estimator and has widely been used as a performance measure in parameter estimation [19]. The vector parameter CRLB places a bound on the variance of each element. The CRLB for the *j*-th parameter of the position estimation vector $\hat{\theta }$ is defined by:

(9) $$\begin{array}{c} \mathrm{CRLB}\left({\left[\hat{\theta }\right]}_{j}\right)={\left[F(\hat{\theta })\right]}_{j,j}^{-1} \end{array}$$

where F is the FIM.

Without loss of generality, assume that the measurement errors are all Gaussian distributions with zero mean error and not relevant:

(10) $$\begin{array}{c} n_{m,n}^{\rho }∼N\left(0,\sigma_{\rho ,m,n}^{2}\right),\;n_{k,n}^{r}∼N\left(0,\sigma_{r,k,n}^{2}\right)\\ n_{k,n}^{\phi }∼N\left(0,\sigma_{\phi ,k,n}^{2}\right),\;n_{k,n}^{\alpha }∼N\left(0,\sigma_{\alpha ,k,n}^{2}\right) \end{array}$$

The derivation of CRLB for collaborative positioning is divided into two cases: the GNSS collaboration with only AOA measurements and both types of relative measurement.

### 3.1. Only AOA Measurements

If the relative distance measurements are not used in collaborative positioning, the available measurement set can be indicated as $t^{\left(1\right)}=\left\{\rho_{m,n},\phi_{k,n},\alpha_{k,n}\right\}$. According to the assumption in Equation (10), the conditional probability density function $p\left\{t^{\left(1\right)}|\theta \right\}$ can be derived as:

(11) $$\begin{array}{c} \begin{array}{cc} p\left\{t^{\left(1\right)}|\theta \right\} & =\frac{1}{\prod_{n=1}^{N}\left(\prod_{m}\left(\sqrt{2\pi }\sigma_{\rho ,m,n}\right)\prod_{k}\left(2\pi \sigma_{\phi ,k,n}\sigma_{\alpha ,k,n}\right)\right)}\\ & \times \exp\left\{\begin{array}{c} -\sum_{n=1}^{N}\sum_{m}\frac{1}{2\sigma_{\rho ,m,n}^{2}}{\left[f_{m,n}^{\rho }\left(\theta \right)\right]}^{2}-\sum_{n=1}^{N}\sum_{k}\frac{1}{2\sigma_{\phi ,k,n}^{2}}{\left[f_{k,n}^{\phi }\left(\theta \right)\right]}^{2}-\sum_{n=1}^{N}\sum_{k}\frac{1}{2\sigma_{\alpha ,k,n}^{2}}{\left[f_{k,n}^{\alpha }\left(\theta \right)\right]}^{2} \end{array}\right\} \end{array} \end{array}$$

where:

(12) $$\begin{array}{cc} f_{m,n}^{\rho }\left(\theta \right) & =\rho_{m,n}-∥p_{m}^{s}-p_{n}^{u}∥-b_{n}^{u}\\ f_{k,n}^{\phi }\left(\theta \right) & =\phi_{k,n}-\mathrm{atan}2\left(y_{k}^{u}-y_{n}^{u},x_{k}^{u}-x_{n}^{u}\right)\\ f_{k,n}^{\alpha }\left(\theta \right) & =\alpha_{k,n}-\mathrm{acos}\left(\frac{z_{k}^{u}-z_{n}^{u}}{d_{k,n}^{u}}\right) \end{array}$$

The elements of FIM are defined by:

(13) $$\begin{array}{c} {\left[F^{\left(1\right)}(\hat{\theta })\right]}_{j,l}=-E\left[\frac{\partial^{2}\ln p\left\{t^{\left(1\right)}|\theta \right\}}{\partial \theta_{j}\partial \theta_{l}}\right],\;j,l=1,2,....,4N \end{array}$$

where the log-likelihood function $\ln p\left\{t^{\left(1\right)}|\theta \right\}$ can be derived as:

(14) $$\begin{array}{c} \ln p\left\{t^{\left(1\right)}|\theta \right\}=C-\frac{1}{2}\sum_{n=1}^{N}\sum_{m}\frac{1}{\sigma_{\rho ,m,n}^{2}}{\left[f_{m,n}^{\rho }\left(\theta \right)\right]}^{2}-\frac{1}{2}\sum_{n=1}^{N}{\sum_{k}\frac{1}{\sigma_{\phi ,k,n}^{2}}\left[f_{k,n}^{\phi }\left(\theta \right)\right]}^{2}-\frac{1}{2}\sum_{n=1}^{N}{\sum_{k}\frac{1}{\sigma_{\alpha ,k,n}^{2}}\left[f_{k,n}^{\alpha }\left(\theta \right)\right]}^{2} \end{array}$$

It can be observed from Equations (13) and (14) that the dimension of FIM is 4N×4N, and the form of FIM can be written as:

(15) $$\begin{array}{c} F^{\left(1\right)}=F_{\rho }+F_{\phi }+F_{\alpha } \end{array}$$

The first term $F_{\rho }$, representing the pseudo-range measurements’ contribution, is expressed as:

(16) $$\begin{array}{cc} F_{\rho } & =G_{\rho }^{T}W_{\rho }G_{\rho } \end{array}$$

where:

(17) $$\begin{array}{c} G_{\rho }=\left[\begin{array}{cccc} G_{\rho ,1} & 0 & \cdots & 0\\ 0 & G_{\rho ,2} & \cdots & 0\\ \vdots & \vdots & \ddots & \vdots \\ 0 & 0 & \cdots & G_{\rho ,N} \end{array}\right],\;W_{\rho }=\left[\begin{array}{cccc} W_{\rho ,1} & 0 & \cdots & 0\\ 0 & W_{\rho ,2} & \cdots & 0\\ \vdots & \vdots & \ddots & \vdots \\ 0 & 0 & \cdots & W_{\rho ,N} \end{array}\right] \end{array}$$

For user #*n*, $G_{\rho ,n}$ represents the effects of visible GNSS satellites and $W_{\rho ,n}$ indicates the weights of observation. If there is no visible satellites for user #*n*, the matrix $G_{\rho ,n}^{T}W_{\rho ,n}G_{\rho ,n}$ is equal to a zero matrix that has the dimension 4×4. Otherwise, the two matrices can be expressed as:

(18) $$\begin{array}{c} G_{\rho ,n}=\left[\begin{array}{c} \beta_{m,n}^{\rho }\\ \vdots \end{array}\right],\;W_{\rho ,n}=\mathrm{diag}\left\{\frac{1}{\sigma_{\rho ,m,n}^{2}}\right\} \end{array}$$

where:

(19) $$\begin{array}{c} \beta_{m,n}^{\rho }=\left[\begin{array}{cccc} \frac{\partial f_{m,n}^{\rho }\left(\theta \right)}{\partial x_{n}^{u}} & \frac{\partial f_{m,n}^{\rho }\left(\theta \right)}{\partial y_{n}^{u}} & \frac{\partial f_{m,n}^{\rho }\left(\theta \right)}{\partial z_{n}^{u}} & \frac{\partial f_{m,n}^{\rho }\left(\theta \right)}{\partial b_{n}^{u}} \end{array}\right]=\left[\begin{array}{cccc} \frac{x_{m}^{s}-x_{n}^{u}}{d_{m,n}^{s}} & \frac{y_{m}^{s}-y_{n}^{u}}{d_{m,n}^{s}} & \frac{z_{m}^{s}-z_{n}^{u}}{d_{m,n}^{s}} & -1 \end{array}\right] \end{array}$$

The AOA collaborative part $F_{\phi }$ and $F_{\alpha }$ can be expressed as:

(20) $$\begin{array}{c} F_{\phi }=G_{\phi }^{T}W_{\phi }G_{\phi },\;F_{\alpha }=G_{\alpha }^{T}W_{\alpha }G_{\alpha } \end{array}$$

where:

(21) $$\begin{array}{cc} G_{\phi } & =\left[\begin{array}{c} G_{\phi ,1}\\ \vdots \\ G_{\phi ,N} \end{array}\right],W_{\phi }=\left[\begin{array}{cccc} W_{\phi ,1} & 0 & \cdots & 0\\ 0 & W_{\phi ,2} & \cdots & 0\\ \vdots & \vdots & \ddots & \vdots \\ 0 & 0 & \cdots & W_{\phi ,N} \end{array}\right]\\ G_{\alpha } & =\left[\begin{array}{c} G_{\alpha ,1}\\ \vdots \\ G_{\alpha ,N} \end{array}\right],W_{\alpha }=\left[\begin{array}{cccc} W_{\alpha ,1} & 0 & \cdots & 0\\ 0 & W_{\alpha ,2} & \cdots & 0\\ \vdots & \vdots & \ddots & \vdots \\ 0 & 0 & \cdots & W_{\alpha ,N} \end{array}\right] \end{array}$$

Similarly, for user #*n*, $G_{\phi ,n}$ and $G_{\alpha ,n}$ represent the effects of AOA measurements and $W_{\phi ,n}$ and $W_{\alpha ,n}$ indicate the weights of corresponding observation. They can be expanded as:

(22) $$\begin{array}{cc} G_{\phi ,n} & =\left[\begin{array}{ccccc} 0 & \overset{k}{\overset{︷}{{\beta^{\prime }}_{k,n}^{\phi }}} & 0 & \overset{n}{\overset{︷}{\beta_{k,n}^{\phi }}} & 0\\ \vdots & \vdots & \vdots & \vdots & \vdots \end{array}\right],\;W_{\phi ,n}=\mathrm{diag}\left\{\frac{1}{\sigma_{\phi ,k,n}^{2}}\right\}\\ G_{\alpha ,n} & =\left[\begin{array}{ccccc} 0 & \overset{k}{\overset{︷}{{\beta^{\prime }}_{k,n}^{\alpha }}} & 0 & \overset{n}{\overset{︷}{\beta_{k,n}^{\alpha }}} & 0\\ \vdots & \vdots & \vdots & \vdots & \vdots \end{array}\right],\;W_{\alpha ,n}=\mathrm{diag}\left\{\frac{1}{\sigma_{\alpha ,k,n}^{2}}\right\} \end{array}$$

where:

(23) $$\begin{array}{c} \begin{array}{cc} \beta_{k,n}^{\phi } & =\left[\begin{array}{cccc} \frac{\partial f_{k,n}^{\phi }\left(\theta \right)}{\partial x_{n}^{u}} & \frac{\partial f_{k,n}^{\phi }\left(\theta \right)}{\partial y_{n}^{u}} & \frac{\partial f_{k,n}^{\phi }\left(\theta \right)}{\partial z_{n}^{u}} & \frac{\partial f_{k,n}^{\phi }\left(\theta \right)}{\partial b_{n}^{u}} \end{array}\right]=\left[\begin{array}{cccc} \frac{y_{k}^{u}-y_{n}^{u}}{{\left(y_{k}^{u}-y_{n}^{u}\right)}^{2}+{\left(x_{k}^{u}-x_{n}^{u}\right)}^{2}} & -\frac{x_{k}^{u}-x_{n}^{u}}{{\left(y_{k}^{u}-y_{n}^{u}\right)}^{2}+{\left(x_{k}^{u}-x_{n}^{u}\right)}^{2}} & 0 & 0 \end{array}\right]\\ {\beta^{\prime }}_{k,n}^{\phi } & =\left[\begin{array}{cccc} \frac{\partial f_{k,n}^{\phi }\left(\theta \right)}{\partial x_{k}^{u}} & \frac{\partial f_{k,n}^{\phi }\left(\theta \right)}{\partial y_{k}^{u}} & \frac{\partial f_{k,n}^{\phi }\left(\theta \right)}{\partial z_{k}^{u}} & \frac{\partial f_{k,n}^{\phi }\left(\theta \right)}{\partial b_{k}^{u}} \end{array}\right]=-\beta_{k,n}^{\phi } \end{array} \end{array}$$

and:

(24) $$\begin{array}{cc} \beta_{k,n}^{\alpha } & =\left[\begin{array}{cccc} \frac{\partial f_{k,n}^{\alpha }\left(\theta \right)}{\partial x_{n}^{u}} & \frac{\partial f_{k,n}^{\alpha }\left(\theta \right)}{\partial y_{n}^{u}} & \frac{\partial f_{k,n}^{\alpha }\left(\theta \right)}{\partial z_{n}^{u}} & \frac{\partial f_{k,n}^{\alpha }\left(\theta \right)}{\partial b_{n}^{u}} \end{array}\right]\\ & =\left[\begin{array}{cccc} -\frac{\left(z_{k}^{u}-z_{n}^{u}\right)\left(x_{k}^{u}-x_{n}^{u}\right)}{{\left(d_{k,n}^{u}\right)}^{3}\sqrt{1-{\left(\frac{z_{k}^{u}-z_{n}^{u}}{d_{k,n}^{u}}\right)}^{2}}} & -\frac{\left(z_{k}^{u}-z_{n}^{u}\right)\left(y_{k}^{u}-y_{n}^{u}\right)}{{\left(d_{k,n}^{u}\right)}^{3}\sqrt{1-{\left(\frac{z_{k}^{u}-z_{n}^{u}}{d_{k,n}^{u}}\right)}^{2}}} & -\frac{{\left(z_{k}^{u}-z_{n}^{u}\right)}^{2}-{\left(d_{k,n}^{u}\right)}^{2}}{{\left(d_{k,n}^{u}\right)}^{3}\sqrt{1-{\left(\frac{z_{k}^{u}-z_{n}^{u}}{d_{k,n}^{u}}\right)}^{2}}} & 0 \end{array}\right]\\ {\beta^{\prime }}_{k,n}^{\alpha } & =\left[\begin{array}{cccc} \frac{\partial f_{k,n}^{\alpha }\left(\theta \right)}{\partial x_{k}^{u}} & \frac{\partial f_{k,n}^{\alpha }\left(\theta \right)}{\partial y_{k}^{u}} & \frac{\partial f_{k,n}^{\alpha }\left(\theta \right)}{\partial z_{k}^{u}} & \frac{\partial f_{k,n}^{\alpha }\left(\theta \right)}{\partial b_{k}^{u}} \end{array}\right]=-\beta_{k,n}^{\alpha } \end{array}$$

### 3.2. Both Distance and AOA Measurements

If both distance measurements and AOA measurements are used in collaborative positioning, the available measurement sets are indicated as $t^{\left(2\right)}=\left\{\rho_{m,n},r_{k,n},\phi_{k,n},\alpha_{k,n}\right\}$. Similarly, according to the assumption in Equation (10), the conditional probability density function $p\left\{t^{\left(2\right)}|\theta \right\}$ can be derived as:

(25) $$\begin{array}{cc} p\left\{t^{\left(2\right)}|\theta \right\} & =\frac{1}{\prod_{n=1}^{N}\left(\prod_{m}\left(\sqrt{2\pi }\sigma_{\rho ,m,n}\right)\prod_{k}\left({\left(2\pi \right)}^{\frac{3}{2}}\sigma_{r,k,n}\sigma_{\phi ,k,n}\sigma_{\alpha ,k,n}\right)\right)}\\ & \times \exp\left\{\begin{array}{c} -\sum_{n=1}^{N}\sum_{m}\frac{1}{2\sigma_{\rho ,m,n}^{2}}{\left[f_{m,n}^{\rho }\left(\theta \right)\right]}^{2}-\sum_{n=1}^{N}\sum_{k}\frac{1}{2\sigma_{r,k,n}^{2}}{\left[f_{k,n}^{r}\left(\theta \right)\right]}^{2}\\ -\sum_{n=1}^{N}\sum_{k}\frac{1}{2\sigma_{\phi ,k,n}^{2}}{\left[f_{k,n}^{\phi }\left(\theta \right)\right]}^{2}-\sum_{n=1}^{N}\sum_{k}\frac{1}{2\sigma_{\alpha ,k,n}^{2}}{\left[f_{k,n}^{\alpha }\left(\theta \right)\right]}^{2} \end{array}\right\} \end{array}$$

Compared to the collaboration with only AOA measurements, the FIM is expanded and has the form of four parts:

(26) $$\begin{array}{c} F^{\left(2\right)}=F_{r}+F_{\rho }+F_{\phi }+F_{\alpha } \end{array}$$

where:

(27) $$\begin{array}{cc} F_{i} & =G_{i}^{T}W_{i}G_{i},\;i\in \left\{\rho ,r,\phi ,\alpha \right\} \end{array}$$

The added term $F_{r}$ representing the distance measurements’ contribution and the matrices $G_{r}$ and $W_{r}$ are derived as:

(28) $$\begin{array}{c} G_{r}=\left[\begin{array}{c} G_{r,1}\\ \vdots \\ G_{r,N} \end{array}\right],\;W_{r}=\left[\begin{array}{cccc} W_{r,1} & 0 & \cdots & 0\\ 0 & W_{r,2} & \cdots & 0\\ \vdots & \vdots & \ddots & \vdots \\ 0 & 0 & \cdots & W_{r,N} \end{array}\right] \end{array}$$

They can be expanded as:

(29) $$\begin{array}{c} G_{r,n}=\left[\begin{array}{ccccc} 0 & \overset{k}{\overset{︷}{{\beta^{\prime }}_{k,n}^{r}}} & 0 & \overset{n}{\overset{︷}{\beta_{k,n}^{r}}} & 0\\ \vdots & \vdots & \vdots & \vdots & \vdots \end{array}\right],\;W_{r,n}=\mathrm{diag}\left\{\frac{1}{\sigma_{r,k,n}^{2}}\right\} \end{array}$$

where:

(30) $$\begin{array}{cc} \beta_{k,n}^{r} & =\left[\begin{array}{cccc} \frac{\partial f_{k,n}^{r}\left(\theta \right)}{\partial x_{n}^{u}} & \frac{\partial f_{k,n}^{r}\left(\theta \right)}{\partial y_{n}^{u}} & \frac{\partial f_{k,n}^{r}\left(\theta \right)}{\partial z_{n}^{u}} & \frac{\partial f_{k,n}^{r}\left(\theta \right)}{\partial b_{n}^{u}} \end{array}\right]=\left[\begin{array}{cccc} -\frac{x_{k}^{u}-x_{n}^{u}}{d_{k,n}^{u}} & -\frac{y_{k}^{u}-y_{n}^{u}}{d_{k,n}^{u}} & -\frac{z_{k}^{u}-z_{n}^{u}}{d_{k,n}^{u}} & 0 \end{array}\right]\\ {\beta^{\prime }}_{k,n}^{r} & =\left[\begin{array}{cccc} \frac{\partial f_{k,n}^{r}\left(\theta \right)}{\partial x_{n}^{u}} & \frac{\partial f_{k,n}^{r}\left(\theta \right)}{\partial y_{n}^{u}} & \frac{\partial f_{k,n}^{r}\left(\theta \right)}{\partial z_{n}^{u}} & \frac{\partial f_{k,n}^{r}\left(\theta \right)}{\partial b_{n}^{u}} \end{array}\right]=-\beta_{k,n}^{r} \end{array}$$

The above results Equations (15) and (26) allow one to compute $F^{\left(1\right)}$ and $F^{\left(2\right)}$ for a given collaborative network configuration and by inverting Equation (9), to express the CRLB, respectively. Therefore, the CRLB for the coordinate estimates are:

(31) $$\begin{array}{c} \mathrm{CRLB}\left({\hat{x}}_{n}^{u}\right)={\left[F^{\left(i\right)}(\hat{\theta })\right]}_{4n+1,4n+1}^{-1},\;\mathrm{CRLB}\left({\hat{y}}_{n}^{u}\right)={\left[F^{\left(i\right)}(\hat{\theta })\right]}_{4n+2,4n+2}^{-1}\\ \mathrm{CRLB}\left({\hat{z}}_{n}^{u}\right)={\left[F^{\left(i\right)}(\hat{\theta })\right]}_{4n+3,4n+3}^{-1},\;\mathrm{CRLB}\left({\hat{b}}_{n}^{u}\right)={\left[F^{\left(i\right)}(\hat{\theta })\right]}_{4n+4,4n+4}^{-1} \end{array}$$

where i∈{1,2} and n=1,2,...N.

## 4. GNSS Collaborative Positioning Algorithm

Making use of AOA measurements or both types of measurement, the cost functions are respectively defined as:

(32) $$\begin{array}{c} J^{\left(1\right)}\left(\theta \right)=\sum_{i=1}^{N}\left[\begin{array}{c} \sum_{m}\frac{{\left[f_{m,n}^{\rho }\left(\theta \right)\right]}^{2}}{\sigma_{\rho ,m,n}^{2}}+\sum_{k}\left(\frac{{\left[f_{k,n}^{\phi }\left(\theta \right)\right]}^{2}}{\sigma_{\phi ,k,n}^{2}}+\frac{{\left[f_{k,n}^{\alpha }\left(\theta \right)\right]}^{2}}{\sigma_{\alpha ,k,n}^{2}}\right) \end{array}\right] \end{array}$$

or:

(33) $$\begin{array}{c} J^{\left(2\right)}\left(\theta \right)=\sum_{i=1}^{N}\left[\begin{array}{c} \sum_{m}\frac{{\left[f_{m,n}^{\rho }\left(\theta \right)\right]}^{2}}{\sigma_{\rho ,m,n}^{2}}+\sum_{k}\frac{{\left[f_{k,n}^{r}\left(\theta \right)\right]}^{2}}{\sigma_{r,k,n}^{2}}+\sum_{k}\frac{{\left[f_{k,n}^{\phi }\left(\theta \right)\right]}^{2}}{\sigma_{\phi ,k,n}^{2}}+\sum_{k}\frac{{\left[f_{k,n}^{\alpha }\left(\theta \right)\right]}^{2}}{\sigma_{\alpha ,k,n}^{2}} \end{array}\right] \end{array}$$

The positions of users are estimated by minimizing the aforementioned cost functions:

(34) $$\begin{array}{c} \hat{\theta }=\arg\min\left\{J^{\left(i\right)}\left(\theta \right)\right\},\;i\in \left\{1,2\right\} \end{array}$$

There are a number of optimization algorithms that can be used to solve the minimization problems in Equation (34) [19]. In this paper, the WLS algorithm is used in combination with the Newton iterative method; thus, it is worth giving a brief description of the algorithm as follows.

The first order Taylor expansion is used to linearize the nonlinear measurements. For user #*n*, the (small) position coordinate estimation errors and the receiver clock bias error are denoted by $\Delta x_{n}^{u}$, $\Delta y_{n}^{u}$, $\Delta z_{n}^{u}$ and $\Delta b_{n}^{u}$.

### 4.1. Only AOA Measurements

Different from the GNSS standalone positioning, for collaboration with AOA measurements, the measurement sets $t^{\left(1\right)}$ are used for GNSS collaborative positioning in this subsection.

By using the first order Taylor expansion, the estimation error in pseudo-range measurements can be approximated as:

(35) $$\begin{array}{cc} \Delta \rho_{m,n} & \approx -\frac{\partial f_{m,n}^{\rho }\left(\theta \right)}{\partial x_{n}^{u}}\Delta x_{n}^{u}-\frac{\partial f_{m,n}^{\rho }\left(\theta \right)}{\partial y_{n}^{u}}\Delta y_{n}^{u}-\frac{\partial f_{m,n}^{\rho }\left(\theta \right)}{\partial z_{n}^{u}}\Delta z_{n}^{u}-\frac{\partial f_{m,n}^{\rho }\left(\theta \right)}{\partial b_{n}^{u}}\Delta b_{n}^{u}\\ & =-\beta_{m,n}^{\rho }\Delta p_{n}^{u} \end{array}$$

where:

(36) $$\begin{array}{cc} \Delta p_{n}^{u} & ={\left[\Delta x_{n}^{u},\Delta y_{n}^{u},\Delta z_{n}^{u},\Delta b_{n}^{u}\right]}^{T} \end{array}$$

In the same way, the estimation error in AOA measurements can be approximated as:

(37) $$\begin{array}{cc} \Delta \phi_{k,n} & \approx -\frac{\partial f_{k,n}^{\phi }\left(\theta \right)}{\partial x_{k}^{u}}\Delta x_{k}^{u}-\frac{\partial f_{k,n}^{\phi }\left(\theta \right)}{\partial y_{k}^{u}}\Delta y_{k}^{u}-\frac{\partial f_{k,n}^{\phi }\left(\theta \right)}{\partial x_{n}^{u}}\Delta x_{n}^{u}-\frac{\partial f_{k,n}^{\phi }\left(\theta \right)}{\partial y_{n}^{u}}\Delta y_{n}^{u}\\ & =\beta_{k,n}^{\phi }\Delta p_{k}^{u}-\beta_{k,n}^{\phi }\Delta p_{n}^{u}\\ \Delta \alpha_{k,n} & \approx -\frac{\partial f_{k,n}^{\alpha }\left(\theta \right)}{\partial x_{k}^{u}}\Delta x_{k}^{u}-\frac{\partial f_{k,n}^{\alpha }\left(\theta \right)}{\partial y_{k}^{u}}\Delta y_{k}^{u}-\frac{\partial f_{k,n}^{\alpha }\left(\theta \right)}{\partial z_{k}^{u}}\Delta z_{k}^{u}\\ & \;-\frac{\partial f_{k,n}^{\alpha }\left(\theta \right)}{\partial x_{n}^{u}}\Delta x_{n}^{u}-\frac{\partial f_{k,n}^{\alpha }\left(\theta \right)}{\partial y_{n}^{u}}\Delta z_{n}^{u}-\frac{\partial f_{k,n}^{\alpha }\left(\theta \right)}{\partial y_{n}^{u}}\Delta z_{n}^{u}\\ & =\beta_{k,n}^{\alpha }\Delta p_{k}^{u}-\beta_{k,n}^{\alpha }\Delta p_{n}^{u} \end{array}$$

where:

(38) $$\begin{array}{cc} \Delta p_{k}^{u} & ={\left[\Delta x_{k}^{u},\Delta y_{k}^{u},\Delta z_{k}^{u},\Delta b_{k}^{u}\right]}^{T} \end{array}$$

Equations (35) and (37) respectively give the error of the pseudo-range and AOA measurements as a function of the positional and clock bias errors. However, the positioning algorithm requires the estimates of the positional and clock bias errors as a function of the pseudo-range and AOA measurement errors. For the convenience of analysis, all of the observational equations based on Equations (35) and (37) can be expressed in a compact form as:

(39) $$\begin{array}{c} G^{\left(1\right)}\Delta \theta \approx v^{\left(1\right)} \end{array}$$

where:

(40) $$\begin{array}{c} G^{\left(1\right)}=\left[\begin{array}{c} G_{\rho }\\ G_{\phi }\\ G_{\alpha } \end{array}\right],v^{\left(1\right)}=\left[\begin{array}{c} v_{\rho }\\ v_{\phi }\\ v_{\alpha } \end{array}\right] \end{array}$$

$v^{\left(1\right)}$ indicates the residual errors of each measurement:

(41) $$\begin{array}{cc} v_{\rho } & =\left[\begin{array}{c} v_{\rho ,1}\\ v_{\rho ,2}\\ \vdots \\ v_{\rho ,N} \end{array}\right],v_{\rho ,n}=\left[\begin{array}{c} -\Delta \rho_{m,n}\\ \vdots \end{array}\right],m\in M_{n}\\ v_{\phi } & =\left[\begin{array}{c} v_{\phi ,1}\\ \vdots \\ v_{\phi ,N} \end{array}\right],v_{\phi ,n}=\left[\begin{array}{c} -\Delta \phi_{k,n}\\ \vdots \end{array}\right],k\in K_{n}\\ v_{\alpha } & =\left[\begin{array}{c} v_{\alpha ,1}\\ \vdots \\ v_{\alpha ,N} \end{array}\right],v_{\alpha ,n}=\left[\begin{array}{c} -\Delta \alpha_{k,n}\\ \vdots \end{array}\right],k\in K_{n} \end{array}$$

### 4.2. Both Distance and AOA Measurements

With both distance and AOA measurements, the measurement sets $t^{\left(2\right)}$ are used here. Similarly, by using the first order Taylor expansion, the estimation error in distance measurements can be approximated as:

(42) $$\begin{array}{cc} \Delta r_{m,n} & \approx -\frac{\partial f_{k,n}^{r}\left(\theta \right)}{\partial x_{n}^{u}}\Delta x_{n}^{u}-\frac{\partial f_{k,n}^{r}\left(\theta \right)}{\partial y_{n}^{u}}\Delta y_{n}^{u}-\frac{\partial f_{k,n}^{r}\left(\theta \right)}{\partial z_{n}^{u}}\Delta z_{n}^{u}-\frac{\partial f_{k,n}^{r}\left(\theta \right)}{\partial b_{n}^{u}}\Delta b_{n}^{u}\\ & =\beta_{k,n}^{r}\Delta p_{k}^{u}-\beta_{k,n}^{r}\Delta p_{n}^{u} \end{array}$$

All of the observational equations based on Equations (35), (37) and (42) can be expressed in a compact form as:

(43) $$\begin{array}{c} G^{\left(2\right)}\Delta \theta \approx v^{\left(2\right)} \end{array}$$

where:

(44) $$\begin{array}{c} G^{\left(2\right)}=\left[\begin{array}{c} G_{\rho }\\ G_{\phi }\\ G_{\alpha }\\ G_{r} \end{array}\right],v^{\left(2\right)}=\left[\begin{array}{c} v_{\rho }\\ v_{\alpha }\\ v_{\phi }\\ v_{r} \end{array}\right] \end{array}$$

Similarly, $v^{\left(2\right)}$ indicates the residual errors of each measurement and $v_{r}$ can be expressed as:

(45) $$\begin{array}{cc} v_{r} & =\left[\begin{array}{c} v_{r,1}\\ \vdots \\ v_{r,N} \end{array}\right],v_{r,n}=\left[\begin{array}{c} -\Delta r_{k,n}\\ \vdots \end{array}\right],k\in K_{n} \end{array}$$

Noting that after first order Taylor expansion, the original set of a nonlinear observation equations turns into a set of linear equations, and furthermore, if the rank of $G^{\left(1\right)}$ or $G^{\left(2\right)}$ is 4N, which means the matrix is column full rank, then a WLS solution for Δθ can be produced:

(46) $$\begin{array}{c} \Delta \hat{\theta }={\left({\left(G^{\left(i\right)}\right)}^{T}W^{\left(i\right)}G^{\left(i\right)}\right)}^{-1}{\left(G^{\left(i\right)}\right)}^{T}W^{\left(i\right)}v^{\left(i\right)} \end{array}$$

where i∈{1,2} and $W^{\left(i\right)}$ is the weighting matrix, which can be expressed as:

(47) $$\begin{array}{cc} W^{\left(1\right)} & =\left[\begin{array}{ccc} W_{\rho } & 0 & 0\\ 0 & W_{\phi } & 0\\ 0 & 0 & W_{\alpha } \end{array}\right],\;W^{\left(2\right)}=\left[\begin{array}{cccc} W_{\rho } & 0 & 0 & 0\\ 0 & W_{\phi } & 0 & 0\\ 0 & 0 & W_{\alpha } & 0\\ 0 & 0 & 0 & W_{r} \end{array}\right] \end{array}$$

In reality, the WLS algorithm described above is typically used in combination with the Newton iterative method. The unknowns in $\Delta \hat{\theta }$ can be obtained by the above calculation, which are used to update the results:

(48) $$\begin{array}{c} {\hat{\theta }}^{\left(q+1\right)}={\hat{\theta }}^{\left(q\right)}+\Delta \hat{\theta } \end{array}$$

where *q* is the iterative count.

After the iteration, $∥\Delta \hat{\theta }∥$ is calculated and, in general, compared to a preset threshold to determine the convergence of Newton iteration algorithm. If $∥\Delta \hat{\theta }∥$ is less than the preset threshold, this means the iterative process has converged and the results can be returned. If not, the process should return to the linearization step and repeat the iterative calculation.

## 5. Performance Analysis

As previously mentioned, the goal of collaboration with AOA measurements is to enhance positioning accuracy and availability for each user. In this section, the theoretical analysis of GNSS collaborative positioning accuracy is presented, including the impacts of various factors and the performance comparison with the positioning without AOA measurements.

### 5.1. Performance with AOA Measurements

**Proposition 1.**  *By adding AOA measurements, the CRLB of each user are always less than or equal to the previous CRLB*.

**Proof of Proposition 1.**  Firstly, the two parts are GNSS standalone positioning and collaborative positioning with only AOA measurements. Assume that each user can complete GNSS standalone positioning, which means the FIM $F_{\rho }$ is an invertible matrix. According to Equations (17)–(19), the matrix $F_{\rho }$ can be written as:

(49) $$\begin{array}{c} F_{\rho }=\left[\begin{array}{cccc} F_{\rho ,1} & 0 & \cdots & 0\\ 0 & F_{\rho ,2} & \cdots & 0\\ \vdots & \vdots & \ddots & \vdots \\ 0 & 0 & \cdots & F_{\rho ,N} \end{array}\right] \end{array}$$

where:

(50) $$\begin{array}{c} F_{\rho ,i}=G_{\rho ,i}^{T}W_{\rho ,i}G_{\rho ,i},\;i=1,2,...,N \end{array}$$

Thus, for user #*n*, the CRLB of GNSS standalone positioning can be expressed as:

(51) $$\begin{array}{c} \mathrm{CRLB}\left({\hat{\theta }}_{j}^{n}\right)={\left[F_{\rho }\right]}_{4n+j,4n+j}^{-1},\;j=1,2,3,4 \end{array}$$

where ${\hat{\theta }}^{n}={\left[x_{n}^{u},y_{n}^{u},z_{n}^{u},b_{n}^{u}\right]}^{T}$.

Then, if the AOA measurements are added for collaboration, the FIM is $F^{\left(1\right)}$. A new matrix is defined $F_{t}=F_{\phi }+F_{\alpha }$; thus, $F^{\left(1\right)}=F_{\rho }+F_{t}$ and $F_{t}$ can be expanded as:

(52) $$\begin{array}{c} F_{t}=G_{t}^{T}W_{t}G_{t} \end{array}$$

where:

(53) $$\begin{array}{c} G_{t}=\left[\begin{array}{c} G_{\phi }\\ G_{\alpha } \end{array}\right],W_{t}=\left[\begin{array}{cc} W_{\phi } & 0\\ 0 & W_{\alpha } \end{array}\right] \end{array}$$

Based on the knowledge of matrix analysis [20], it can be obtained by using the Woodbury formula:

(54) $$\begin{array}{cc} {\left(F^{\left(1\right)}\right)}^{-1} & ={\left(F_{\rho }+F_{t}\right)}^{-1}={\left(F_{\rho }+G_{t}^{T}W_{t}G_{t}\right)}^{-1}\\ & =F_{\rho }^{-1}-F_{\rho }^{-1}G_{t}^{T}{\left(W_{t}^{-1}+G_{t}F_{\rho }^{-1}G_{t}^{T}\right)}^{-1}G_{t}F_{\rho }^{-1} \end{array}$$

Since $F_{\rho }$ is a symmetric positive definite matrix, $F_{\rho }^{-1}$ is also a symmetric positive definite matrix. By applying eigenvalues decomposition, $F_{\rho }^{-1}$ is decomposed of eigenvectors and eigenvalues:

(55) $$\begin{array}{c} F_{\rho }^{-1}=Q_{\rho }^{T}\Lambda_{\rho }Q_{\rho } \end{array}$$

in which $\Lambda_{\rho }=\mathrm{diag}\left\{\lambda_{\rho ,1},\lambda_{\rho ,2},...,\lambda_{\rho ,4N}\right\}$ is a diagonal matrix, which is constructed by the positive eigenvalues of $F_{\rho }^{-1}$, and $Q_{\rho }$ is the corresponding orthogonal matrix. The matrix $G_{t}^{T}Q_{\rho }$ is written in rows as:

(56) $$\begin{array}{c} G_{t}^{T}Q_{\rho }=\left[\begin{array}{c} a_{1}\\ a_{2}\\ \vdots \\ a_{4N} \end{array}\right] \end{array}$$

Thus, it can be obtained that:

(57) $$\begin{array}{c} G_{t}F_{\rho }^{-1}G_{t}^{T}=\sum_{j=1}^{4N}\lambda_{\rho ,j}a_{j}^{T}a_{j} \end{array}$$

It can be known from the construction of $G_{t}F_{\rho }^{-1}G_{t}^{T}$ that it is at least a symmetric positive semidefinite matrix. Similarly, by applying eigenvalue decomposition, $G_{t}F_{\rho }^{-1}G_{t}^{T}$ is decomposed as:

(58) $$\begin{array}{c} G_{t}F_{\rho }^{-1}G_{t}^{T}=Q_{t}^{T}\Lambda_{t}Q_{t} \end{array}$$

where $\Lambda_{t}$ is the diagonal matrix with non-negative eigenvalues and $Q_{t}$ is the corresponding orthogonal matrix. Meanwhile, the matrix $W_{t}^{-1}$ is a diagonal positive definite matrix; thus, it can be derived:

(59) $$\begin{array}{c} {\left(W_{t}^{-1}+G_{t}F_{\rho }^{-1}G_{t}^{T}\right)}^{-1}=Q_{t}^{T}{\left(W_{t}^{-1}+\Lambda_{t}\right)}^{-1}Q_{t} \end{array}$$

Define $T_{1}=Q_{t}G_{t}F_{\rho }^{-1}$ and $\Lambda_{1}={\left(W_{t}^{-1}+\Lambda_{t}\right)}^{-1}$. It can be seen from the construction that $\Lambda_{1}$ is a diagonal positive definite matrix. Thus, the matrix $X_{1}=T_{1}^{T}\Lambda_{1}T_{1}$ is defined, and it can be rewritten as:

(60) $$\begin{array}{c} X_{1}=\sum_{j=1}^{L}\lambda_{1,j}t_{1,j}^{T}t_{1,j} \end{array}$$

where $\lambda_{1,j}$ is the *j*-th diagonal element of $\Lambda_{1}$, $t_{1,j}$ is the *j*-th row vector of $T_{1}$, $L=\sum_{n=1}^{N}\mathrm{Card}\left(K_{n}\right)$ and $\mathrm{Card}\left(K_{n}\right)$ indicates the number of collaborative users for user #*n*.

Since $\lambda_{1,j}>0$, so the $X_{1}$ is also a at least a symmetric positive semidefinite matrix from the definition. Meanwhile, the matrix $F_{\rho }^{-1}$ is a symmetrical positive matrix. According to the properties of the positive semidefinite matrix, the diagonal elements of the matrix are all non-negative. Therefore, the following Inequation can be obtained from Equation (54):

(61) $$\begin{array}{c} h_{j,j}^{\left(1\right)}=h_{\rho ,j,j}-x_{1,j,j}\leq h_{\rho ,j,j},\;1\leq j\leq 4N \end{array}$$

where $h_{j,j}^{\left(1\right)},h_{\rho ,j,j}$ and $x_{1,j,j}$ are respectively the *j*-th diagonal elements of ${\left(F^{\left(1\right)}\right)}^{-1},F_{\rho }^{-1}$ and $X_{1}$.

If the two parts are GNSS collaborative positioning with distance measurements and both types of measurements, the two FIMs are respectively: $\left(F_{\rho }+F_{r}\right)$ and $F^{\left(2\right)}$. Similar to previous derivation of Inequation (61), the similar result can be obtained:

(62) $$\begin{array}{c} h_{j,j}^{\left(2\right)}\leq h_{j,j}^{\prime },\;1\leq j\leq 4N \end{array}$$

where $h_{j,j}^{\left(2\right)}$ and $h_{j,j}^{\prime }$ are respectively the *j*-th diagonal elements of ${\left(F^{\left(2\right)}\right)}^{-1}$ and ${\left(F_{\rho }+F_{r}\right)}^{-1}$.

All in all, according to the definition of CRLB for each user in Equations (31) and (51), it can be derived from Inequations (61) and (62) that by adding AOA measurements, the CRLB of each user are always less than or equal to the previous CRLB. ☐

In addition, by adding AOA measurements, the positioning availability of each user can also be improved. For user #*n* in GNSS standalone positioning, the number of visible satellites must be great than or equal to four:

(63) $$\begin{array}{c} \mathrm{Card}\left(M_{n}\right)\geq 4 \end{array}$$

Extended to multi-user collaboration with AOA measurements, there is no strict limit on the number of visible satellites. The necessary condition for collaboration with AOA measurements is:

(64) $$\begin{array}{c} \sum_{n=1}^{N}\mathrm{Card}\left(M_{n}\right)+\frac{2\times \sum_{n=1}^{N}\mathrm{Card}\left(K_{n}\right)}{2}\;\overset{\Delta }{=}M+L\geq 4N \end{array}$$

If the condition of Equation (63) is met, the Equation (64) is always fulfilled. However, conversely, if Equation (64) is met, the condition of Equation (63) may not be fulfilled. For example, there are two users in the network, and each user can see three satellites. At this moment, the Equation (63) is not met. However, if the two users can collaborate with each other, it can be derived that:

(65) $$\begin{array}{c} M+L=6+2=8=4N \end{array}$$

which means the positioning availability of each user is improved by collaboration.

### 5.2. The Impact of AOA Measurements Accuracy

**Proposition 2.**  *The CRLB of each user increase with the increasing of AOA measurement errors, and when the AOA measurement errors are big enough, the collaboration does not bring any benefit*.

**Proof of Proposition 2.**  In this subsection, we assume that two different AOA measuring technologies are used, and the corresponding measured variances respectively are $\left\{\sigma_{\phi 1,k,n}^{2},\sigma_{\alpha 1,k,n}^{2}\right\}$ and $\left\{\sigma_{\phi 2,k,n}^{2},\sigma_{\alpha 2,k,n}^{2}\right\}$. Under the two different variances, the two corresponding FIMs are denoted as $\left\{F_{\phi 1},F_{\alpha 1}\right\}$ and $\left\{F_{\phi 2},F_{\alpha 2}\right\}$, respectively.

In the first case, the two parts are GNSS standalone positioning and collaborative positioning with only AOA measurements. Therefore, the following Inequation can be obtained from Equation (54):

(66) $$\begin{array}{c} {\left(F_{1}^{\left(1\right)}\right)}^{-1}-{\left(F_{2}^{\left(1\right)}\right)}^{-1}=T_{1}^{T}\Lambda_{t12}T_{1} \end{array}$$

where:

(67) $$\begin{array}{cc} {\left(F_{1}^{\left(1\right)}\right)}^{-1} & =F_{\rho }+F_{t1}=F_{\rho }+F_{\phi 1}+F_{\alpha 1}\\ {\left(F_{2}^{\left(1\right)}\right)}^{-1} & =F_{\rho }+F_{t2}=F_{\rho }+F_{\phi 2}+F_{\alpha 2} \end{array}$$

and $\Lambda_{\phi 12}$ is a diagonal matrix, which is expressed as:

(68) $$\begin{array}{c} \Lambda_{t12}=\left(W_{t1}^{-1}-W_{t2}^{-1}\right){\left(W_{t1}^{-1}+\Lambda_{t}\right)}^{-1}{\left(W_{t2}^{-1}+\Lambda_{t}\right)}^{-1} \end{array}$$

Denote by $\lambda_{t12,j}$ the *j*-th diagonal element of $\Lambda_{t12}$; thus, it can be derived:

(69) $$\begin{array}{c} {\left(F_{1}^{\left(1\right)}\right)}^{-1}-{\left(F_{2}^{\left(1\right)}\right)}^{-1}=\sum_{j=1}^{L}\lambda_{t12,j}t_{1,j}^{T}t_{1,j} \end{array}$$

If $\sigma_{\phi 1,k,n}^{2}>\sigma_{\phi 2,k,n}^{2}$ and $\sigma_{\alpha 1,k,n}^{2}>\sigma_{\alpha 2,k,n}^{2}$, then it can be obtained that $\lambda_{t12,j}>0$, and the matrix (${\left(F_{1}^{\left(1\right)}\right)}^{-1}-{\left(F_{2}^{\left(1\right)}\right)}^{-1}$) is a symmetric positive semidefinite matrix. According to the properties of positive semidefinite matrix, we get:

(70) $$\begin{array}{c} h_{1,j,j}^{\left(1\right)}\geq h_{2,j,j}^{\left(1\right)},\;1\leq j\leq 4N \end{array}$$

where $h_{1,j,j}^{\left(1\right)}$ and $h_{2,j,j}^{\left(1\right)}$ are the *j*-th diagonal elements of ${\left(F_{1}^{\left(1\right)}\right)}^{-1}$ and ${\left(F_{2}^{\left(1\right)}\right)}^{-1}$, respectively.

Meanwhile, when $\sigma_{\phi ,k,n}^{2}$ and $\sigma_{\alpha ,k,n}^{2}$ are big enough, the matrix $X_{1}$ tends to a zero matrix. Thus, it can be derived from Equation (54) that:

(71) $$\begin{array}{c} {\left(F^{\left(1\right)}\right)}^{-1}=F_{\rho }^{-1}-X_{1}\approx F_{\rho }^{-1} \end{array}$$

If the two parts are GNSS collaborative positioning with distance measurements and collaborative positioning with both distance and AOA measurements, similar results can also be obtained.

According to the definition of CRLB for each user in Equation (31), it can be derived that the CRLB of each user increase with the increasing of AOA measurement errors, and when the measurement errors are big enough, the collaboration does not bring any benefit. ☐

### 5.3. The Impacts of the Number of Users and Collaborative Links

**Proposition 3.**  *If a new collaborative link is added to the user network, the CRLB of each user are always less than or equal to the previous values*.

**Proof of Proposition 3.**  For GNSS collaborative positioning, if a new collaborative link between the *k*-th user and the *n*-th user is added to the user network, the new FIM is defined by $F_{e1}$, and it can be written as:

(72) $$\begin{array}{cc} F_{e1} & =F_{c1}+G_{c}^{T}W_{c}G_{c} \end{array}$$

where:

(73) $$\begin{array}{cc} G_{c} & =\left[\begin{array}{ccccc} 0 & \overset{k}{\overset{︷}{{\beta^{\prime }}_{k,n}^{\phi }}} & 0 & \overset{n}{\overset{︷}{\beta_{k,n}^{\phi }}} & 0\\ 0 & \overset{k}{\overset{︷}{{\beta^{\prime }}_{k,n}^{\alpha }}} & 0 & \overset{n}{\overset{︷}{\beta_{k,n}^{\alpha }}} & 0\\ 0 & \overset{k}{\overset{︷}{{\beta^{\prime }}_{n,k}^{\phi }}} & 0 & \overset{n}{\overset{︷}{\beta_{n,k}^{\phi }}} & 0\\ 0 & \overset{k}{\overset{︷}{{\beta^{\prime }}_{n,k}^{\alpha }}} & 0 & \overset{n}{\overset{︷}{\beta_{n,k}^{\alpha }}} & 0 \end{array}\right],\;W_{c}=\left[\begin{array}{cccc} \frac{1}{\sigma_{\phi ,k,n}^{2}} & 0 & 0 & 0\\ 0 & \frac{1}{\sigma_{\alpha ,k,n}^{2}} & 0 & 0\\ 0 & 0 & \frac{1}{\sigma_{\phi ,n,k}^{2}} & 0\\ 0 & 0 & 0 & \frac{1}{\sigma_{\alpha ,n,k}^{2}} \end{array}\right] \end{array}$$

The matrix $F_{c1}$ equals $F^{\left(1\right)}$ for the collaborative positioning with only AOA measurements and equals $F^{\left(2\right)}$ for the collaborative positioning with distance and AOA measurements. Since the AOA measurements technologies of different users may be different, the variance of measurement errors may not be equal. For instance, $\sigma_{\phi ,k,n}^{2}$ and $\sigma_{\phi ,n,k}^{2}$ may not be equal.

It can be known from the construction that $G_{c}^{T}W_{c}G_{c}$ is a symmetric positive semidefinite matrix. Thus, by using the Woodbury formula, we get:

(74) $$\begin{array}{cc} F_{e1}^{-1} & ={\left(F^{\left(i\right)}+G_{c}^{T}W_{c}G_{c}\right)}^{-1}\\ & =F_{c1}^{-1}-F_{c1}^{-1}G_{c}^{T}{\left(W_{c}^{-1}+G_{c}F_{c1}^{-1}G_{c}^{T}\right)}^{-1}G_{c}F_{c1}^{-1} \end{array}$$

Therefore, the similar derivation and result of Inequation (61) can be obtained:

(75) $$\begin{array}{c} h_{e1,j,j}\leq h_{c1,j,j},\;1\leq j\leq 4N \end{array}$$

where $h_{e1,j,j}$ and $h_{c1,j,j}$ are the *j*-th diagonal elements of matrix $F_{e1}^{-1}$ and $F_{c1}^{-1}$, respectively.

In the physical sense, it does mean if a new collaborative link is added to the user network, the CRLB of each user are always less than or equal to the previous values. ☐

**Proposition 4.**  *If a new user and its collaborative links are added to the user network, the CRLB of each user are always less than or equal to the previous values*.

**Proof of Proposition 4.**  It is assumed that a new user #*y* and its collaborative links are added to the user group; the new FIM is defined by $F_{e2}$, and it can be written as:

(76) $$\begin{array}{c} F_{e2}=F_{c2}+G_{t,y}^{T}W_{t,y}G_{t,y} \end{array}$$

where:

(77) $$\begin{array}{cc} F_{c2} & =\left[\begin{array}{cc} F^{\left(i\right)} & 0\\ 0 & F_{\rho ,y} \end{array}\right],\;i=1,2\\ G_{t,y} & =\left[\begin{array}{c} G_{\phi ,y}\\ G_{\alpha ,y} \end{array}\right],W_{t,y}=\left[\begin{array}{cc} W_{\phi ,y} & 0\\ 0 & W_{\alpha ,y} \end{array}\right] \end{array}$$

It can be known from the construction that $G_{t,y}^{T}W_{t,y}G_{t,y}$ is a symmetric positive semidefinite matrix. By using the Woodbury formula, we get:

(78) $$\begin{array}{cc} F_{e2}^{-1} & ={\left(F_{c2}+G_{t,y}^{T}W_{t,y}G_{t,y}\right)}^{-1}\\ & =F_{c2}^{-1}-F_{c2}^{-1}G_{t,y}^{T}{\left(W_{t,y}^{-1}+G_{t,y}F_{c2}^{-1}G_{t,y}^{T}\right)}^{-1}G_{t,y}F_{c2}^{-1} \end{array}$$

Therefore, the similar derivation and result of Inequation (61) can be obtained:

(79) $$\begin{array}{c} h_{e2,j,j}\leq h_{c2,j,j},\;1\leq j\leq 4\left(N+1\right) \end{array}$$

where $h_{e2,j,j}$ and $h_{c2,j,j}$ are the *j*-th diagonal elements of matrix $F_{e2}^{-1}$ and $F_{c2}^{-1}$, respectively. This means that if a new user and its collaborative links are added to the user network, the CRLB of each user are always less than or equal to the previous values. ☐

## 6. Simulation Results

Based on the performance analysis in the previous section, the next simulation tests are carried out to verify the analysis results in different scenarios.

### 6.1. Performance with AOA Measurements

**Figure 2:**
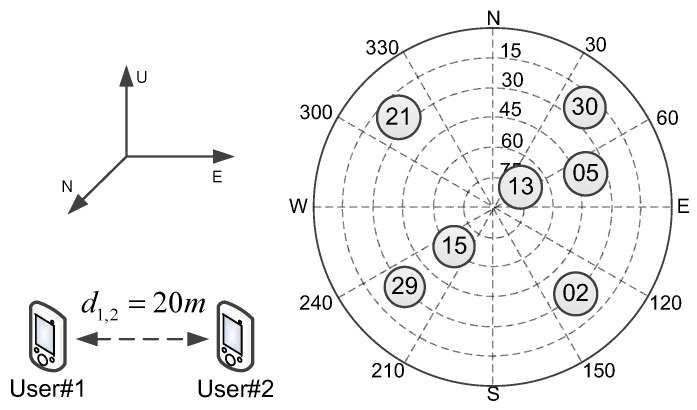
User distribution and skyplot of the outdoor simulation scenario.

Figure 2 gives the distribution of visible GNSS satellites in the following tests. It can be seen that the simulation scenario contains seven satellites, and the distribution of them is relatively uniform.

There are two users in the collaborative user group, and assume that each user can see all of the satellites. Table 1 and Table 2 give the users’ and satellites’ position in east-north-up (ENU) coordinates, respectively. For most collaborative applications, such as a motorcade drives through urban canyons and overpasses and tunnels, the 10-m level between collaborative users is very common distance. Thus, the distance of 20 m is chosen as the simulation parameter. The user #1 is chosen as the process center in the following simulation tests. Of course, a dedicated process center can also be used.

During the following simulation tests, the standard deviations of measurements are set, respectively, to [21,22]:

(80) $$\begin{array}{cc} \sigma_{\rho ,m,n}=7.1\;m & ,\;\sigma_{r,k,n}=1\;m\\ \sigma_{\phi ,k,n}=4{}^{\circ} & ,\;\sigma_{\alpha ,k,n}=4{}^{\circ} \end{array}$$

**Figure 3:**
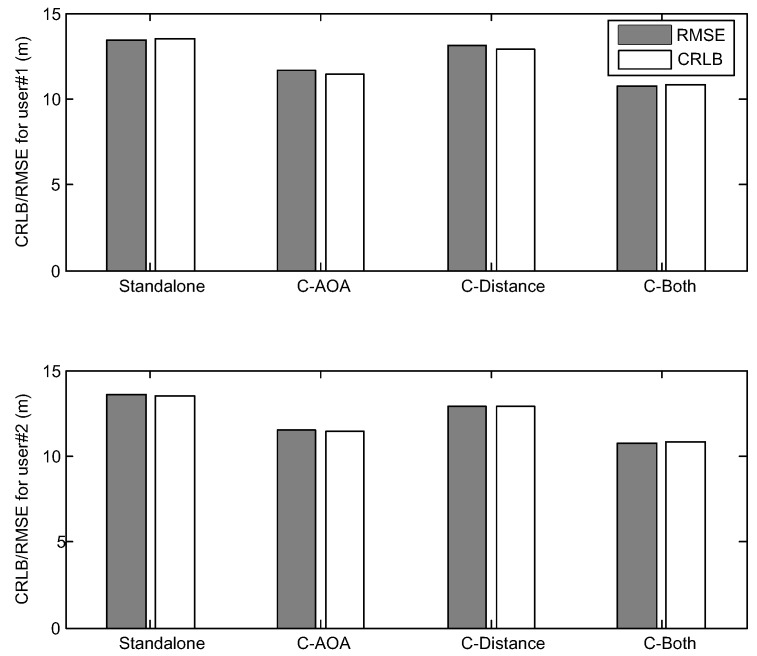
The Cramer–Rao lower bound (CRLB)/RMSE of the outdoor simulation scenario.

Based on the above parameters, the CRLB and root-mean square error (RMSE) of the GNSS standalone WLS and collaborative WLS algorithms are obtained by using Monte Carlo simulation over 10,000 tests, and the results are shown in Figure 3. The performance measure is the RMSE of the user three-dimensional position estimation. Additionally, the same user and satellite position configurations are used to compute the average three-dimensional CRLB, which is defined as:

(81) $$\begin{array}{c} \sigma_{CRLB-3D}^{n}=\sqrt{\sum_{j=1}^{3}{\left[\mathrm{CRLB}\left({\hat{\theta }}_{j}^{n}\right)\right]}^{2}} \end{array}$$

For analytical and visual convenience, the collaborative positioning with the pseudo-range and AOA measurement is abbreviated as C-AOA; the collaborative positioning with the pseudo-range and distance measurements is abbreviated as C-Distance; and the collaborative positioning with the pseudo-range, distance and AOA measurements is abbreviated as C-Both.

It can be observed from Figure 3 that, by adding AOA measurements, the CRLB and RMSE of each user are always less than or equal to the previous results. Meanwhile, the RMSE results of the WLS algorithms are very close to the CRLB, and the accuracy of two users is almost the same. Thus, in the following simulation tests, the histograms of the RMSE results are drawn together with the CRLB results and are not listed separately.

**Figure 4:**
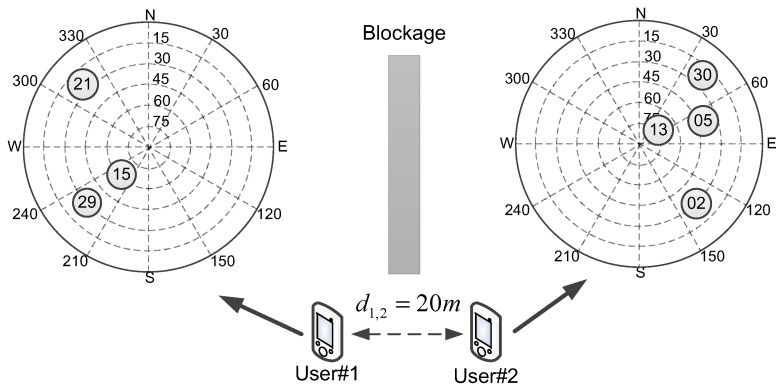
User distribution and skyplot of the blockage simulation scenario.

In the next simulation, it is assumed that each user can only see part of the satellites. Figure 4 shows the blockage simulation scenario.

It can be seen from Figure 4 that, due to the blockage, user #1 can only see the left three satellites and user #2 can see the right four satellites. Because of the lack of visible satellites, user #1 cannot complete GNSS standalone positioning in the simulation. However, for collaborative positioning with the pseudo-range and AOA measurements, it can be calculated that the number of independent measurements is greater than the number of unknowns, that is:

(82) $$\begin{array}{c} M+L=7+2=9\geq 4N \end{array}$$

which means the C-AOA algorithm can be carried out, and the position of user #1 and user #2 can be obtained.

**Figure 5:**
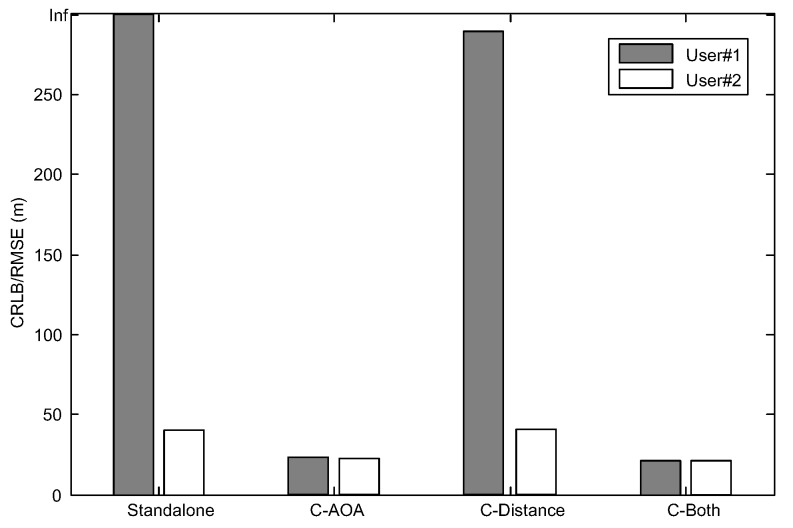
The CRLB/RMSE of the blockage simulation scenario.

Based on previous parameters in Table 1 and Table 2 and Equation (80), the Monte Carlo simulation over 10,000 tests is implemented for this blockage scenario. Figure 5 shows the CRLB and RMSE results of the standalone and collaborative positioning.

It can be clearly seen from Figure 5 that, since the number of visible satellites is less than four, user #1 is unable to complete GNSS standalone positioning. However, for GNSS collaborative positioning, especially for the collaboration with AOA measurements, the collaborative positioning algorithms work, and the performances are significantly improved. Similarly, by adding AOA measurements, the CRLB and RMSE results of user #2 are all less than the previous results, which is consistent with the analysis in Proposition 1 in Section 5.1.

### 6.2. The Impact of AOA Measurements Accuracy

**Figure 6:**
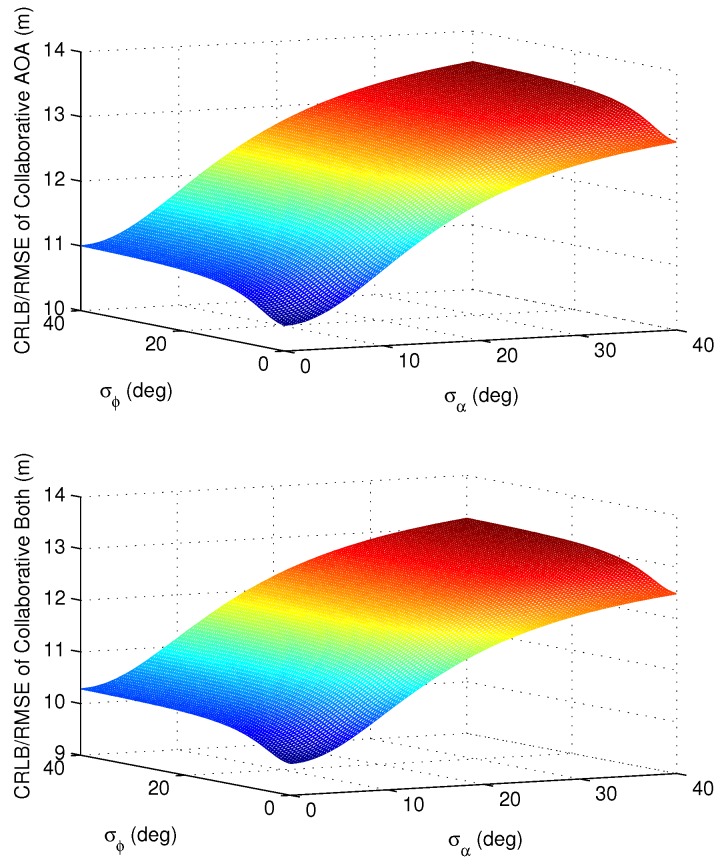
The impact of AOA measurements accuracy for user #1.

Next, a simulation test is carried out to verify the impact of AOA measurement accuracy. Under the scenario introduced in Figure 2, Table 1 and Table 2, Figure 6 shows the accuracy of the CRLB and RMSE derived with respect to the relative azimuth and elevation estimation errors, respectively. The standard deviation of relative distance measurements is fixed as $\sigma_{r}=2$ m.

As shown in Figure 6, for collaboration with AOA and both types of measurements, the CRLB and RMSE of user #1 increase with the increasing of AOA measurement errors, and when the measurement errors are big enough, the collaboration does not bring any benefit. The results of user #2 are almost the same as the results of user #1, so Figure 6 only shows the results of user #1. The simulation results are consistent with the analysis in Proposition 2 in Section 5.2.

With the development of AOA measuring technologies, a variety of technologies can already guarantee that $\sigma_{\phi }$ and $\sigma_{\alpha }$ are always smaller than 10° [10]. For this case, it can be seen that the change of azimuth accuracy has little impact on the positioning performance, while the impact of elevation accuracy is more obvious for the positioning results. This conclusion provides a useful reference for practical applications. It can be observed that the AOA measuring technologies with high azimuth measuring accuracy are not particularly necessary for collaborative applications, and the promotion of elevation measuring accuracy is more critical for collaborative positioning accuracy.

### 6.3. The Impacts of the Number of Users and Collaborative Links

**Figure 7:**
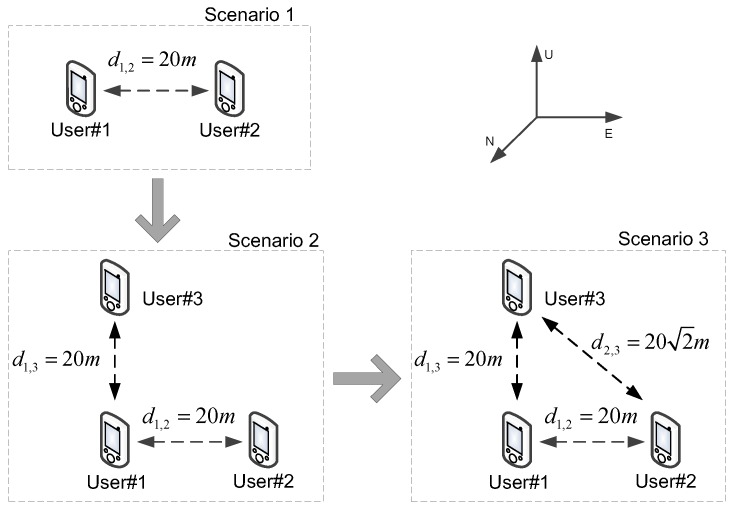
User distribution with new collaborative link and user.

Under the scenario introduced in Figure 2, Table 1 and Table 2 and Equation (80), for Scenario 2, the user #3 and the collaborative link between user #1 and user #3 are added to the user group. Additionally, for Scenario 3, the extra link between user #2 and user #3 is added based on Scenario 2. The ENU coordinates of user #3 are set as ${\left[0,0,20\right]}^{T}$, and Figure 7 shows the user distribution with new user and collaborative links.

**Figure 8:**
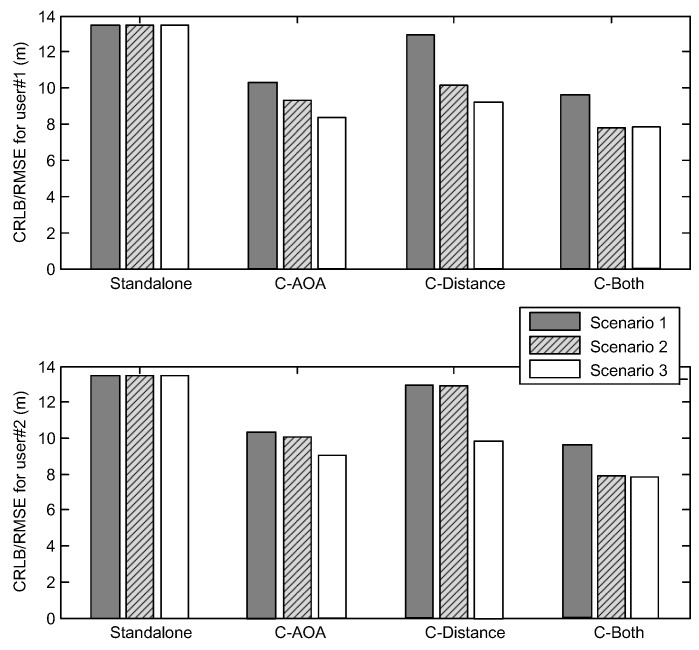
The impact of new collaborative link and user.

Under the three simulation scenarios introduced in Figure 7, Figure 8 shows the simulation results to illustrate the impact of the extra user and links. It can be seen that the CRLB/RMSE of user #1 and user #2 are reduced more than before, which is consistent with the theoretical result in Propositions 3 and 4 in Section 5.3. Meanwhile, from Scenario 1 to Scenario 2, the performance improvement of user #1 is greater than the improvement of user #2 because the additional user #3 has no collaborative link with user #2. From Scenario 2 to Scenario 3, because of the additional link between user #2 and user #3, the performance improvement of user #2 is significant, and the performance improvement of user #1 is relative small. The preliminary results shows that, for each user, the number of collaborative links has a certain influence on the positioning accuracy.

### 6.4. The Impacts of the Relative Location of the User

For GNSS standalone receiver and collaborative positioning receiver with only distance measurements, if processing capability is limited, there is a restriction on the number of measurements that can be obtained and processed. Alternatively, in order to reduce the computational complexity of position determination, the number of measurements can be restricted, and the receiver must select some satellites and users with a good distribution to participate in the calculation [8,23].

**Figure 9:**
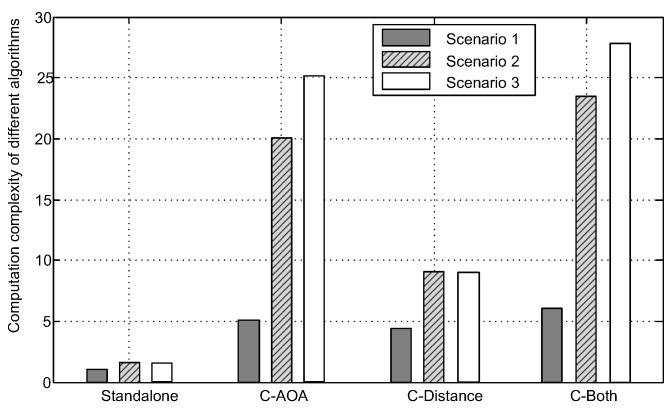
The computation complexity of new collaborative link and user.

Figure 9 shows the amount of calculation for the previous simulation in Figure 8. The computational load is based on the average elapsed time of each collaborative positioning algorithm in Monte Carlo simulation.

Taking the average elapsed time of GNSS standalone positioning algorithm for Scenario 1 as a benchmark, it can be seen from Figure 9 that the average elapsed time of collaborative positioning algorithm with AOA measurements significantly increases with the increase of the amount of users. In addition, compared to the collaborative positioning with distance measurements, the average elapsed time of collaborative positioning with AOA measurements is significantly improved as the user #3 is added to the group. There are several reasons that lead to the increase of computational complexity, mainly including the matrix dimension and average iteration times of the iterative WLS algorithms. Although the collaborative positioning accuracy improves with the increasing of the number of collaborative users, in order to weigh the amount of calculation and performance, it also needs to select a small number of users with which to collaborate. Therefore, for the simulation in Figure 8 and Figure 9, if the processing capability of collaborative process center is limited, maybe we can only implement the collaborative positioning with two users.

**Figure 10:**
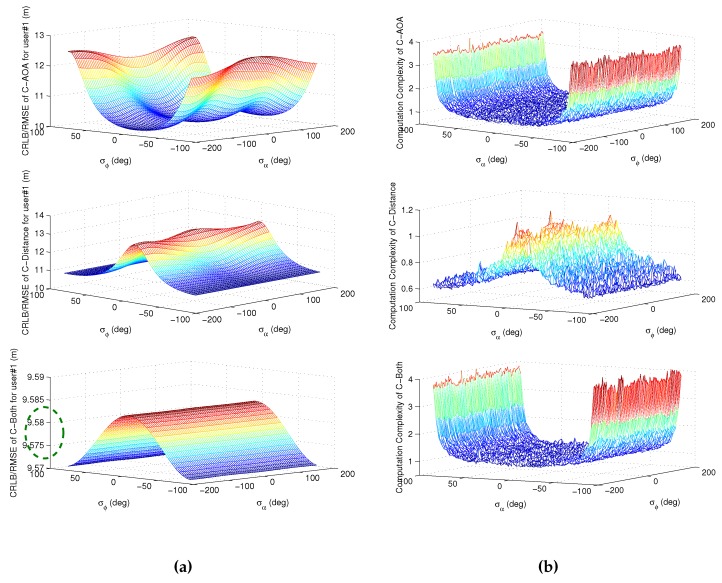
The impact of user distribution on positioning accuracy and computation complexity. (**a**) The positioning accuracy of user #1; (**b**) the average elapsed time of collaborative positioning.

Thus, considering that there are some limitations on increasing the number of users and collaborative links due to power and complexity constraints, the influences of the relative location of the collaborative users need to be discussed in order to choose appropriate neighboring users. The following analyses are focused on the impacts of different relative locations in order to explain the phenomena in Figure 8 and Figure 9. Under the satellite distribution in Figure 2, a collaborative simulation with two users is carried out to test the impact of user distribution. The ENU coordinate of user #1 is fixed to ${\left[0,0,0\right]}^{T}$; the relative distance between user #1 and user #2 is fixed to 20 m; and the coordinate of user #2 depends on the elevation and azimuth angle relative to user #1. Figure 10 shows the simulation results of positioning accuracy and computation complexity.

First, Figure 10a gives the CRLB/RMSE results with the different elevation and azimuth angle of user #2. It can be seen that for collaborative positioning with AOA measurements, user #2 with lower elevation (absolute value) gets the higher positioning accuracy, but for collaborative positioning with distance measurements, the results are just the opposite. However, for collaborative positioning with both types of measurements, it can be seen from the green dashed line that the influence of different azimuth and elevation is very small. Besides, it can also be seen that the influence of azimuth is relatively smaller than that of elevation, and a different azimuth has little impact on the final positioning accuracy.

Then, the computational complexity results of the simulation in Figure 10a are shown in Figure 10b. The computational complexity is based on the average elapsed time of each collaborative positioning algorithm in Monte Carlo simulation over 10,000 tests. The average elapsed time is normalized by the average running time of collaborative positioning with AOA measurements with zero elevation and zero azimuth, which means the computational complexity of two is double the average running time of the collaborative positioning with AOA measurements with zero elevation and zero azimuth. It can be seen that, for collaborative positioning with AOA measurements and both types of measurements, the collaborative user #2 with lower elevation (absolute value) has the lower computational complexity. The main reason is the average iteration times of the iterative WLS algorithms vary with different elevation angles. The higher elevation of user #2 would cause more average iteration times and, thus, lead to more average running time. However, for collaborative positioning with only distance measurements, the influence of a different azimuth and elevation on computation complexity is relatively small. Meanwhile, the similar results of Figure 10a can be obtained, such that the influence of azimuth is relatively smaller than that of elevation.

All in all, it can be seen from Figure 10 that the selection strategy of neighboring users is not the same for different collaborations. For the collaboration with AOA measurements, the neighboring user with lower elevation (absolute value) can obtain both lower positioning error and computational complexity. However, for collaboration with distance measurements, the results are just the opposite, which means the neighboring user with higher elevation (absolute value) should be the one with whom collaboration is preferred.

## 7. Experiment Results

**Figure 11:**
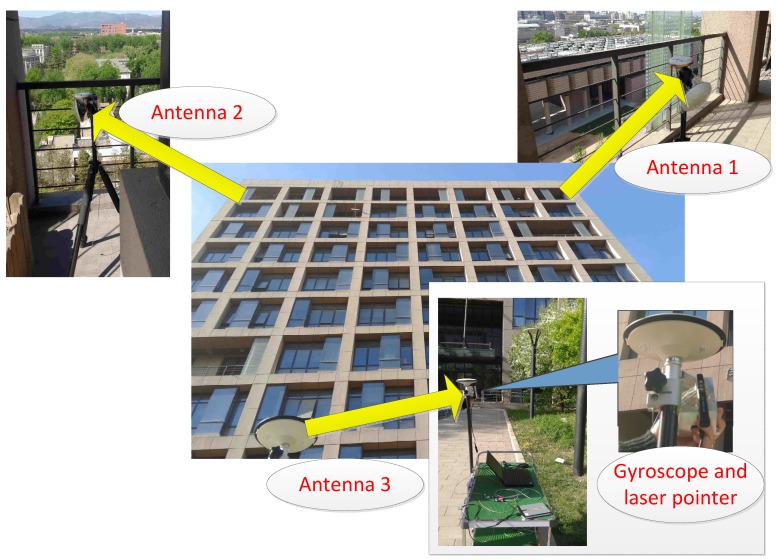
The experiment scenario.

In this section, the derived analytical results are evaluated by using actual signals. The antennas are laid on the balcony and the south side of Weiqing Building of Tsinghua University in Beijing, China, and three u-blox receivers are used to process GNSS signals. Figure 11 shows the actual signal experiment scenario, which contains three users and the AOA measuring equipment.

In this experiment, the position of user #3 is taken as the origin of the reference system, and the Earth Centered Earth Fixed (ECEF) coordinate is ${\left[-2170097.87,4385040.48,4078120.45\right]}^{T}$; the relative positions of the other two users, expressed in ENU coordinates, are shown in Table 3.

**Figure 12:**
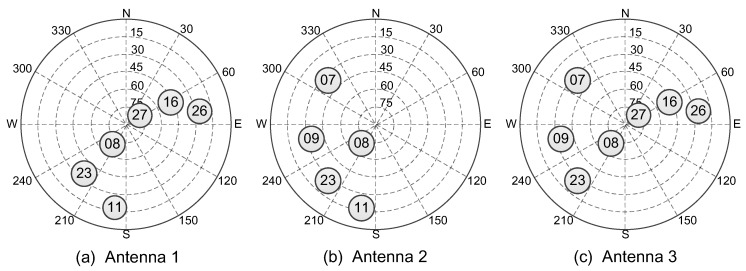
The visible satellites of each antenna.

Figure 12 shows the visible satellites of each antenna during the experiment period. It can be seen that, due to occlusion, the number of visible satellites has decreased for each user, and the poor distribution of the satellites would cause the large dilution of precision (DOP) values and poor positioning accuracy in GNSS standalone positioning.

Generally, the AOA measurements are obtained by applying the antenna arrays, and the measurement accuracy is less than 10°. In this paper, due to the limitation of the experimental conditions, a simpler alternative method has been adopted. A gyroscope and a laser pointer are used to measure the AOA measurements. The laser pointer is bound on a gyroscope around an antenna, and the laser pointer is pointed to another antenna manually. During this process, the AOA measurements can be obtained from the gyroscope, and the measurement accuracy is less than 1°. Due to the simulation results in Figure 6, the impact of the angle measurements accuracy is relatively small when the standard deviations of angle measurements are less than 10°. Thus, the performance of collaborative positioning based on our experimental equipment is similar to the results based on antenna arrays.

**Figure 13:**
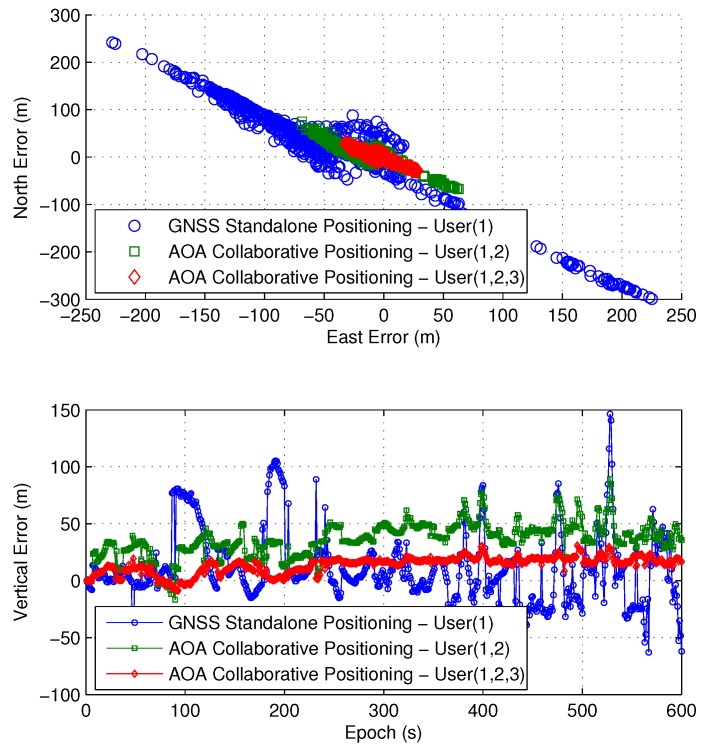
Positioning results of different collaboration methods.

Under this actual experiment scenario, the GNSS collaborative positioning test is carried out with pseudo-range measurements and AOA measurements. For user #1, Figure 13 shows the positioning results of actual data experiment over 600 seconds, and the statistical results are given in Table 4.

It can be seen that, when user #1 works alone in this harsh environment, the positioning error can reach hundreds of meters, which is very large and cannot meet the demand for most GNSS applications. While by collaboration with AOA measurements, the positioning error can be significantly reduced. Meanwhile, the positioning accuracy increases with the increase of the number of users, which is also consistent with the derived analytical results.

## 8. Conclusions

In this paper, the algorithms and CRLB of GNSS collaborative positioning with AOA measurements are proposed. Theoretical analyses are carried out to analyze and verify the collaborative positioning accuracy and the impacts of various factors, which show that the GNSS collaborative positioning accuracy with AOA measurements is always higher than or equal to that without AOA measurements; the accuracy decreased with the increasing of AOA measurements error; the accuracy is always higher than or equal to the previous results if a new communication link or a new user and its collaborative links are added to the user group. Considering that there are limitations on increasing the number of users and collaborative links due to power and complexity constraints, the influences of the relative location of collaborative users are also discussed in order to select appropriate neighboring users. In outdoor and partial blockage scenarios, respectively, simulations are done with several users. Besides, the collaborative positioning experiment is carried out with several GNSS receivers in an actual scenario. Theoretical analysis and experimental results are consistent with the theoretical analysis, and both verify that the positioning performance can be improved by adding AOA measurements to collaboration; also, the results can provide some useful information for the design and testing of the actual collaboration applications.

## Figures and Tables

**Table 1:** The users’ east-north-up (ENU) coordinates of the outdoor scenario.

User Number	East (m)	North (m)	Up (m)
1	0	0	0
2	20	0	0

**Table 2:** The satellites’ ENU coordinates of the outdoor scenario.

Sat Number	East (10^6^ m)	North (10^6^ m)	Up (10^6^ m)
13	2.851289	5.147142	−19.458578
2	−17.322397	13.667608	−7.823561
29	−10.835696	−15.379902	−12.403265
15	−6.490995	−6.722849	18.288367
5	4.833740	14.109308	−15.636070
30	16.311682	16.623783	−6.435868
21	12.874734	−15.017447	−12.187870

**Table 3:** The users’ coordinates of the actual scenario.

User Number	East (m)	North (m)	Under (m)
1	12.4266	20.1483	40.2278
2	−21.2774	17.8867	40.2000
3	0	0	0

**Table 4:** Experimental statistics results.

Positioning Method	East (m)	North (m)	Vertical (m)
GNSS Standalone Positioning: User (1)	84.837	100.745	31.414
AOA Collaborative Positioning: User (1,2)	25.906	24.832	14.994
AOA Collaborative Positioning: User (1,2,3)	12.951	11.938	7.925

## References

[1] Caceres M.A., Sottile F., Garello R. Peer-to-Peer Cooperative Positioning Part II: Hybrid Devices with GNSS & Terrestrial Ranging Capability. http://www.insidegnss.com/auto/julyaug12-WP.pdf.

[2] The European GNSS Agency GNSS Market Report 2015—Issue 4.

[3] Heinrichs G., Mulassano P., Dovis F. A hybrid positioning algorithm for cellular radio networks by using a common rake receiver architecture. Proceedings of the Symposium on Personal, Indoor and Mobile Radio.

[4] Caceres M.A., Sottile F., Garello R., Spirito M.A. Hybrid GNSS-ToA localization and tracking via cooperative unscented Kalman filter. Proceedings of the IEEE 21st International Conference on Personal, Indoor and Mobile Radio Communications (PIMRC) Workshops.

[5] Sottile F., Wymeersch H., Caceres M.A., Spirito M.A. Hybrid GNSS-terrestrial cooperative positioning based on particle filter. Proceedings of the Global Telecommunications Conference (GLOBECOM 2011).

[6] Caceres M.A., Penna F., Wymeersch H., Garello R. (2011). Hybrid cooperative positioning based on distributed belief propagation. IEEE J. Sel. Areas Commun..

[7] Caceres M.A., Sottile F., Garello R., Spirito M.A. (2010). Cramer-Rao Bound for hybrid GNSS-terrestrial cooperative positioning. IEEE Commun. Lett..

[8] Huang B., Yao Z., Cui X., Lu M. (2016). Dilution of Precision Analysis for GNSS Collaborative Positioning. IEEE Trans. Veh. Technol..

[9] Taponecco L., D’Amico A., Mengali U. (2011). Joint TOA and AOA estimation for UWB localization applications. IEEE Trans. Wirel. Commun..

[10] Gavish M., Weiss A.J. (1992). Performance analysis of bearing-only target location algorithms. IEEE Trans. Aerosp. Electron. Syst..

[11] Urruela A., Pages-Zamora A., Riba J. Divide-and-Conquer Based Closed-form Position Estimation for AOA and TDOA Measurements. Proceedings of the IEEE International Conference on Acoustics, Speech and Signal Processing.

[12] Kulakowski P., Vales-Alonso J., Egea-Lopez E., Ludwin W., Garcia-Haro J. (2010). Angle-of-arrival localization based on antenna arrays for wireless sensor networks. Comput. Electr. Eng..

[13] Martinelli A., Siegwart R. Observability analysis for mobile robot localization. Proceedings of the IEEE/RSJ International Conference on Intelligent Robots and Systems.

[14] Xu J., Ma M., Law C.L. AOA cooperative position localization. Proceedings of the IEEE Global Telecommunications Conference.

[15] Sharma R., Quebe S., Beard R.W., Taylor C.N. (2013). Bearing-only cooperative localization. J. Intell. Robot. Syst..

[16] Spiker J.J. (1996). The Global Positioning System: Theory and Application.

[17] Jourdan D.B., Dardari D., Win M.Z. (2008). Position error bound for UWB localization in dense cluttered environments. IEEE Trans. Aerosp. Electron. Syst..

[18] Klukas R., Fattouche M. (1998). Line-of-sight angle of arrival estimation in the outdoor multipath environment. IEEE Trans. Veh. Technol..

[19] Kay S.M. (1995). Estimation Theory. Fundamentals of Statistical Signal Processing.

[20] Horn R.A., Johnson C.R. (2012). Matrix Analysis.

[21] Kaplan E.D., Hegarty J. (2005). Understanding GPS: Principles and Applications.

[22] Zekavat R., Buehrer R.M. (2011). Handbook of Position Location: Theory, Practice and Advances.

[23] Phatak M.S. (2001). Recursive method for optimum GPS satellite selection. IEEE Trans. Aerosp. Electron. Syst..

